# Supplementary figures and images for: The glycosyltransferase POGLUT1 regulates muscle stem cell development and maintenance in mice
Source: PLoS Genet. 2025 Aug 18;21(8):e1011806. doi: 10.1371/journal.pgen.1011806 (PMC12373270; doi:10.1371/journal.pgen.1011806)

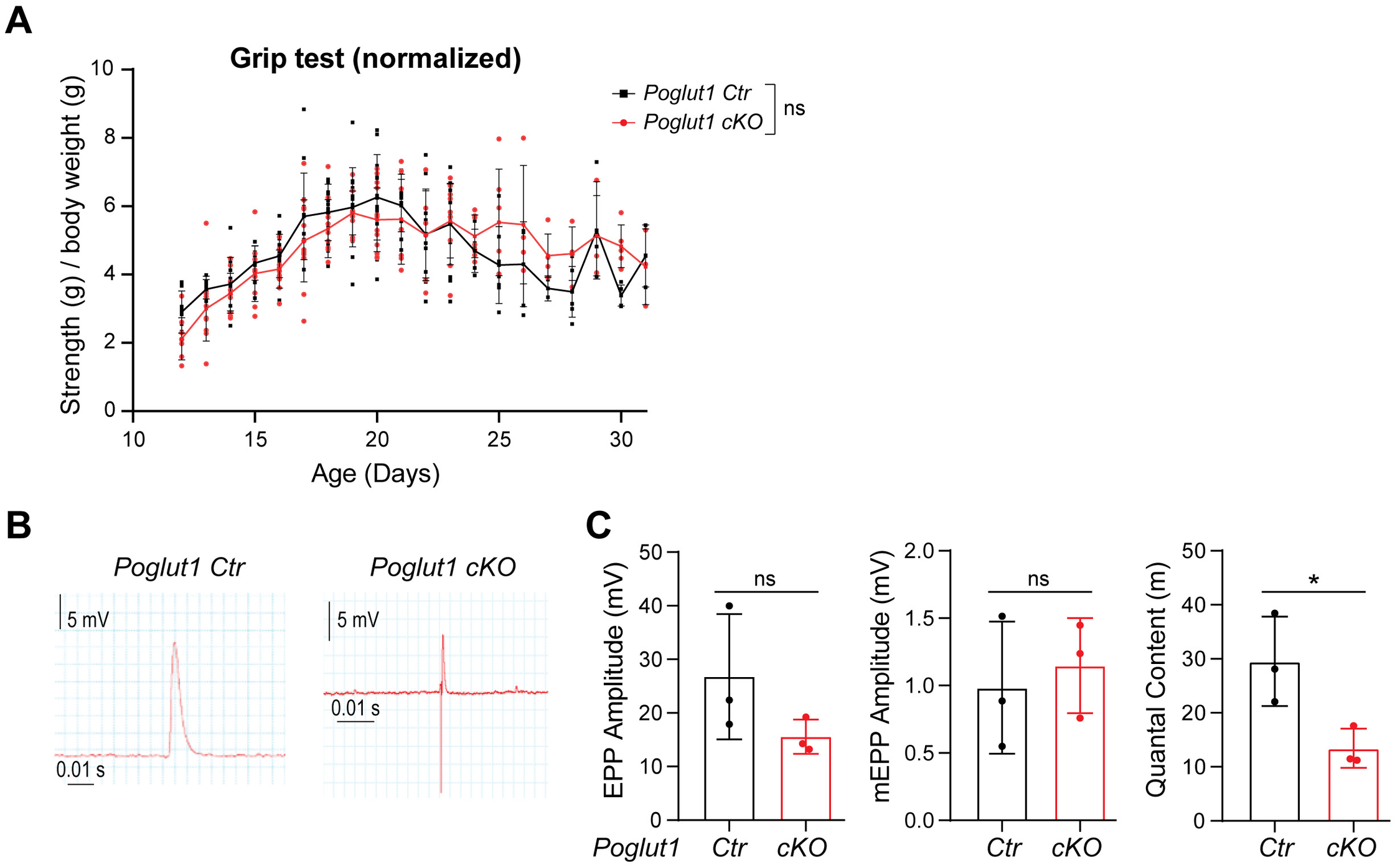

Supplement: S1 Fig — A) Grip test of control and cKO mice normalized by body weight. B) Electrophysiological recordings from LAL muscles at low frequency stimulation (0.5 Hz, 100 s). C) Quantification of the EPP amplitude, miniature EPP (mEPP) amplitude, and the quantal content from control and Poglut1-cKO muscles. The mean mEPPs amplitude showed no significant difference between control and cKO fibers, suggesting comparable spontaneous release. While the mean size of the EPPs showed no significant difference between cKO and control, the quantal content values of cKO were significantly lower than that in control, suggesting an alteration in neurotransmission in cKO mice (Poglut1-Ctr: 3 mice (52 terminals); Poglut1-cKO: 3 mice (33 terminals)). In A and C, each dot represents an animal. Mean±SD is shown. Two-way ANOVA with Šidák’s multiple comparisons test (A); Unpaired t test (C). Row factor (the overall effect of genotype) is shown for A. ns: not significant, *P < 0.05. (TIF) [file pgen.1011806.s001.tif]

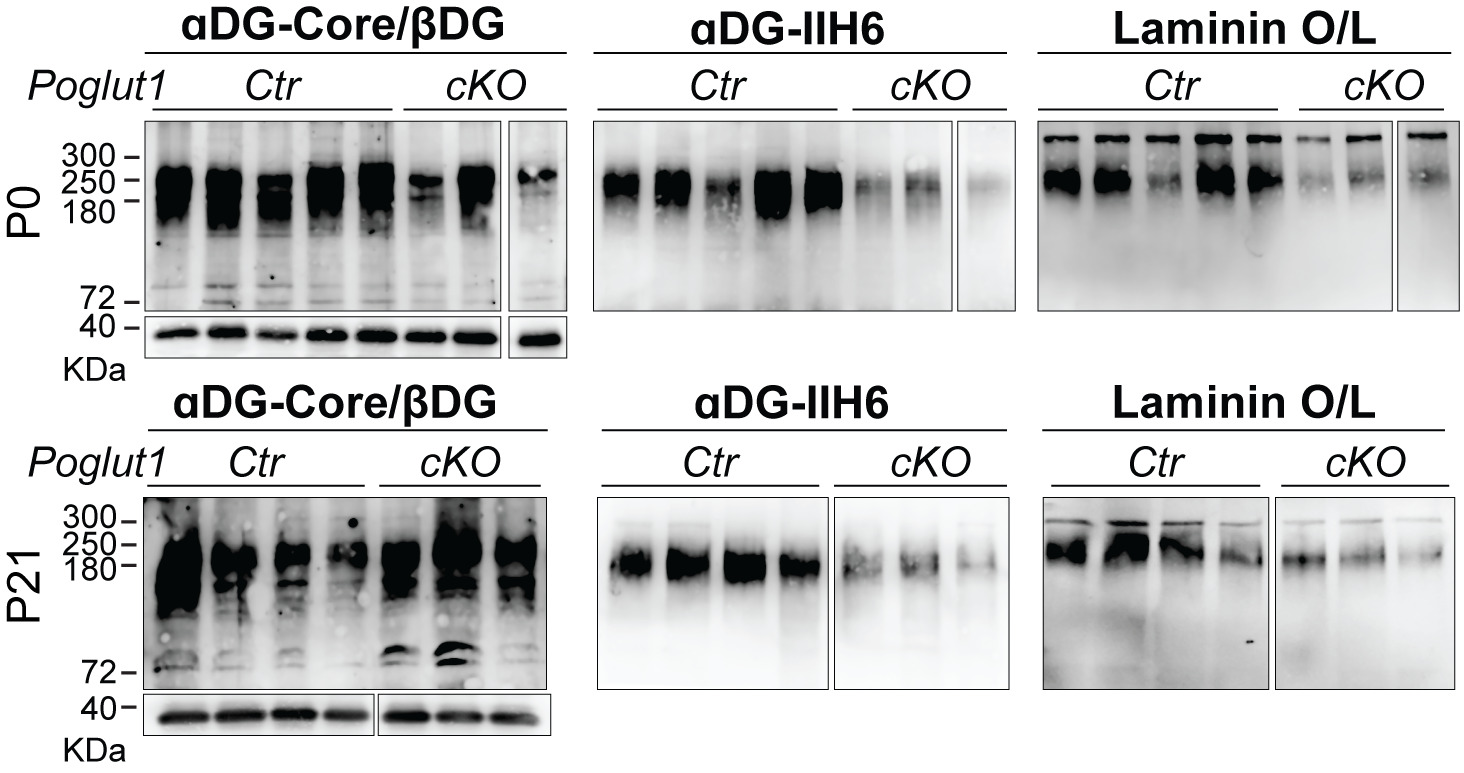

Supplement: S2 Fig — Shown is α-DG glycosylation status in Poglut1-cKO and control mouse muscles at P0 and P20 assessed by western blotting using antibodies against the glycosylated form of α-DG (αDG-IIH6) and the core α-DG, and by laminin overlay assay. An antibody against β-DG was used to assess loading. Wheat germ agglutinin-enriched muscle lysates from control and cKO mice were used. The western blot showed a reduced expression level of glycosylated α-dystroglycan in cKO mice at P0 and P20, as well as a diminished binding activity in the laminin overlay assay when compared to control mice. Each lane represents an individual animal. Boxes mark samples taken from non-adjacent wells of the same gel. (TIF) [file pgen.1011806.s002.tif]

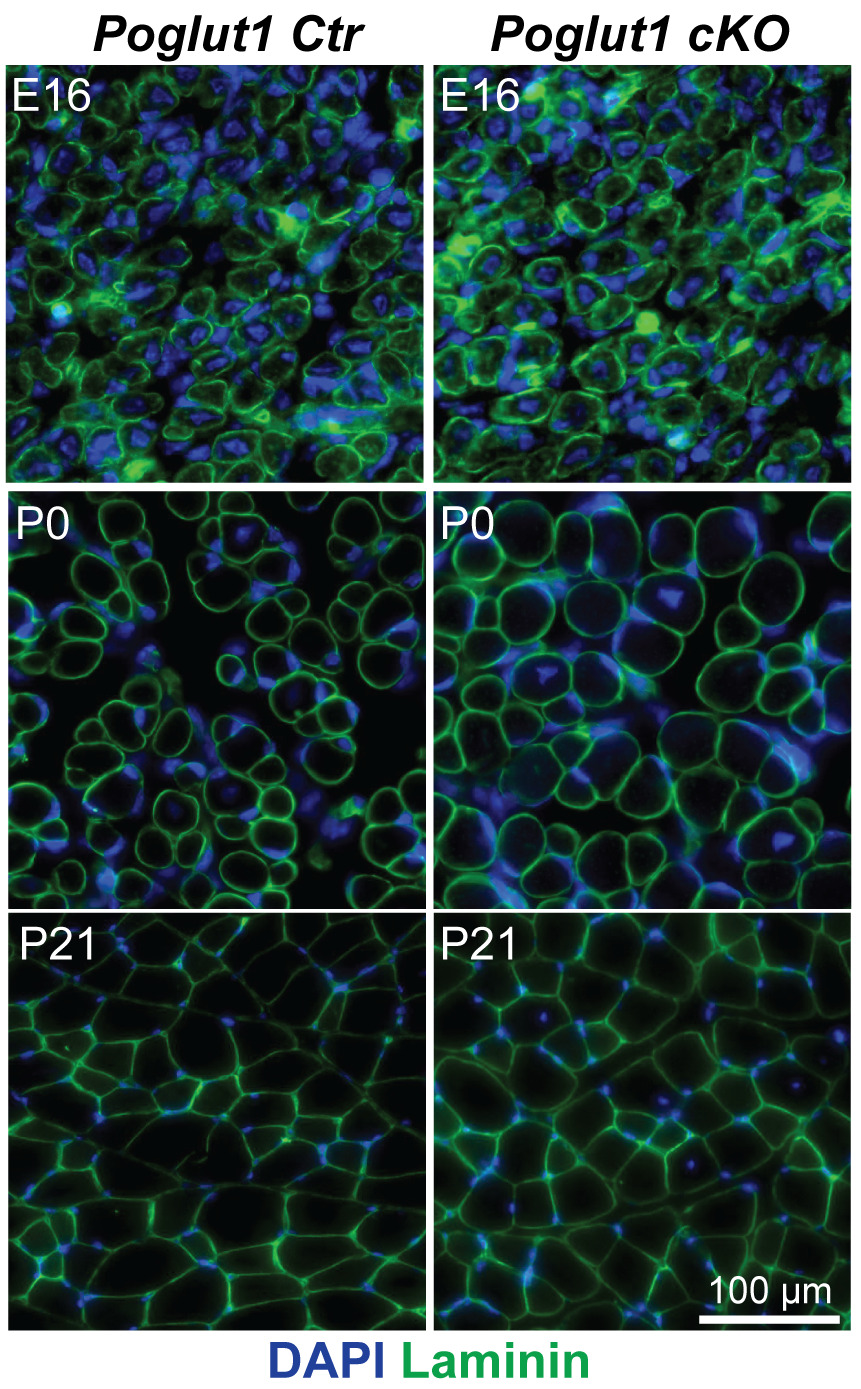

Supplement: S3 Fig — Shown is the DAPI, laminin co-staining of TA muscles from the indicated genotypes at E16, P0, and P21. Note a higher frequency of myofibers with internal nuclei in P0 and P21 Poglut1-cKO muscles compared to sibling controls without Poglut1 deletion (Poglut1+/+, Poglut1flox/flox, or Pax7Cre; Poglut1+/+). Quantification is shown in Fig 3A. (TIF) [file pgen.1011806.s003.tif]

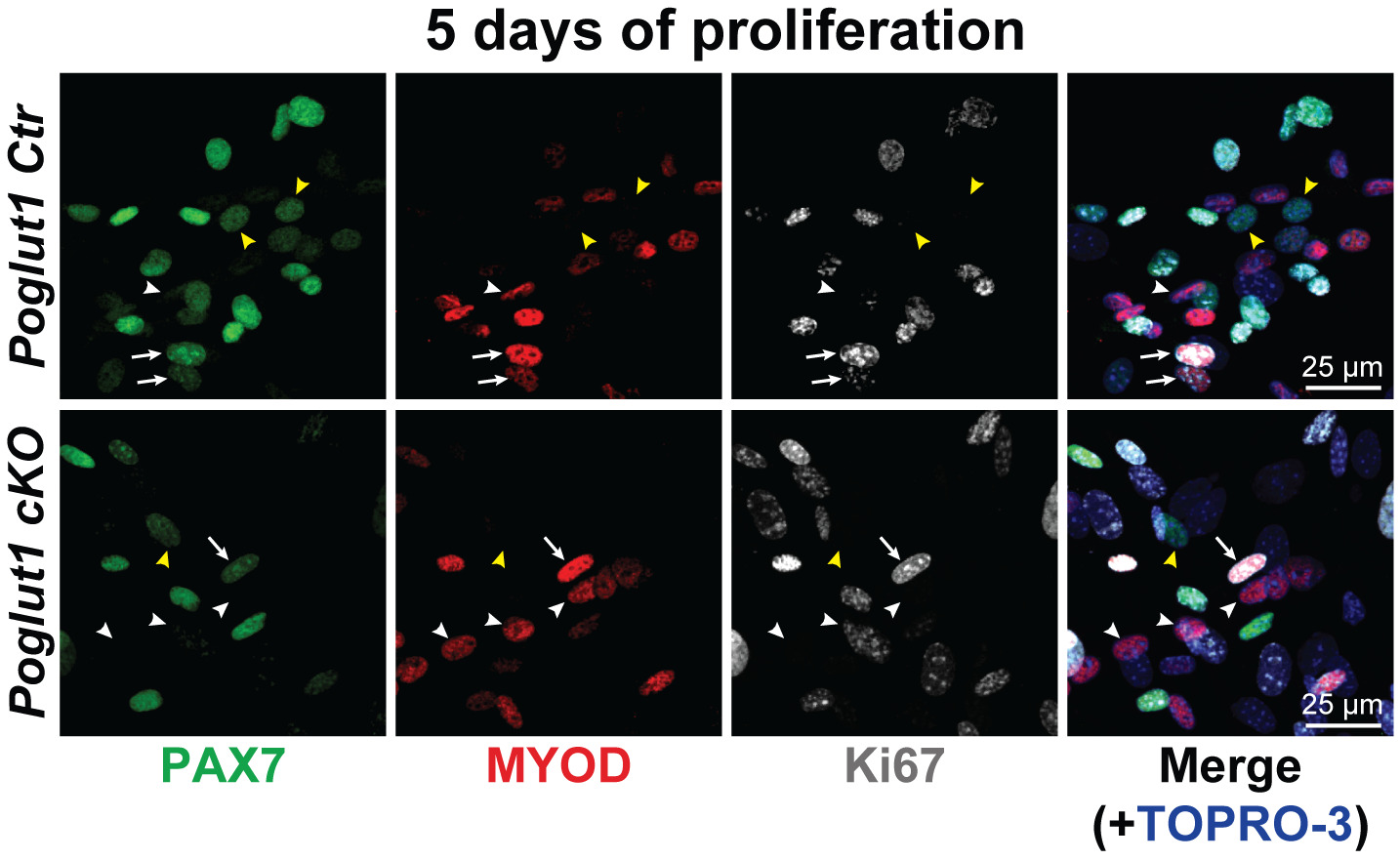

Supplement: S4 Fig — Shown is the PAX7 MYOD Ki67 triple staining of myogenic progenitors isolated from forelimb and hindlimb muscles of P4 Poglut1-cKO and sibling controls without Poglut1 deletion (Poglut1+/+, Poglut1flox/flox, or Pax7Cre; Poglut1+/+) and cultured in proliferation media for five days. TOPRO-3 marks the nuclei. Yellow arrowheads, white arrows and white arrowheads mark examples of quiescent muscle stem cells (PAX7+ MYOD– Ki67–), cycling progenitor cells (PAX7+ MYOD+ Ki67+), and non-cycling precursor cells (PAX7– MYOD+ Ki67–), respectively. Quantifications are shown in Fig 3H. (TIF) [file pgen.1011806.s004.tif]
