# Supplementary material for: The glycosyltransferase POGLUT1 regulates muscle stem cell development and maintenance in mice
Source: PLoS Genet. 2025 Aug 18;21(8):e1011806. doi: 10.1371/journal.pgen.1011806 (PMC12373270; doi:10.1371/journal.pgen.1011806)
Supplement: S5 Fig — Representative MS/MS spectra are shown on the left, and Extracted Ion Chromatograms (EICs) on the right for peptides containing the POGLUT1 consensus site from mouse NOTCH3 EGF repeats. A) Peptide with O-glucose monosaccharide on EGF2. B) Peptide with O-glucose-xylose-xylose trisaccharide on EGF3. C) Peptide with O-glucose-xylose-xylose trisaccharide on EGF9. D) Peptide with O-glucose-xylose-xylose trisaccharide on EGF11. E) Peptide with O-glucose-xylose-xylose trisaccharide on EGF13. F) Peptide with O-glucose-xylose-xylose trisaccharide and O-GlcNAc monosaccharide on EGF15. G) Peptide with O-glucose-xylose-xylose trisaccharide and O-GlcNAc monosaccharide on EGF16. H) Peptide with O-glucose-xylose-xylose trisaccharide and O-fucose-HexNAc-Hexose trisaccharide on EGF17. I) Peptide with O-glucose-xylose-xylose trisaccharide and O-fucose monosaccharide on EGF19. J) Peptide with O-glucose-xylose-xylose trisaccharide and O-fucose monosaccharide on EGF23. K) Peptide with O-glucose monosaccharide on EGF25. L) Peptide with O-glucose-xylose-xylose trisaccharide and O-fucose monosaccharide on EGF29. M) Peptide with O-glucose-xylose-xylose trisaccharide on EGF32. The b- and y-ions are indicated in blue and red font, respectively. ~ indicates ions that lost glucose in the gas phase during fragmentation. Due to the lability of O-glycans in the gas phase during collision, these modifications can fall off which can lead to incorrect annotation of the modified amino acid. However, we can predict the correct position based on the putative consensus sequence for modification by POGLUT1. (PDF) [file pgen.1011806.s005.pdf]

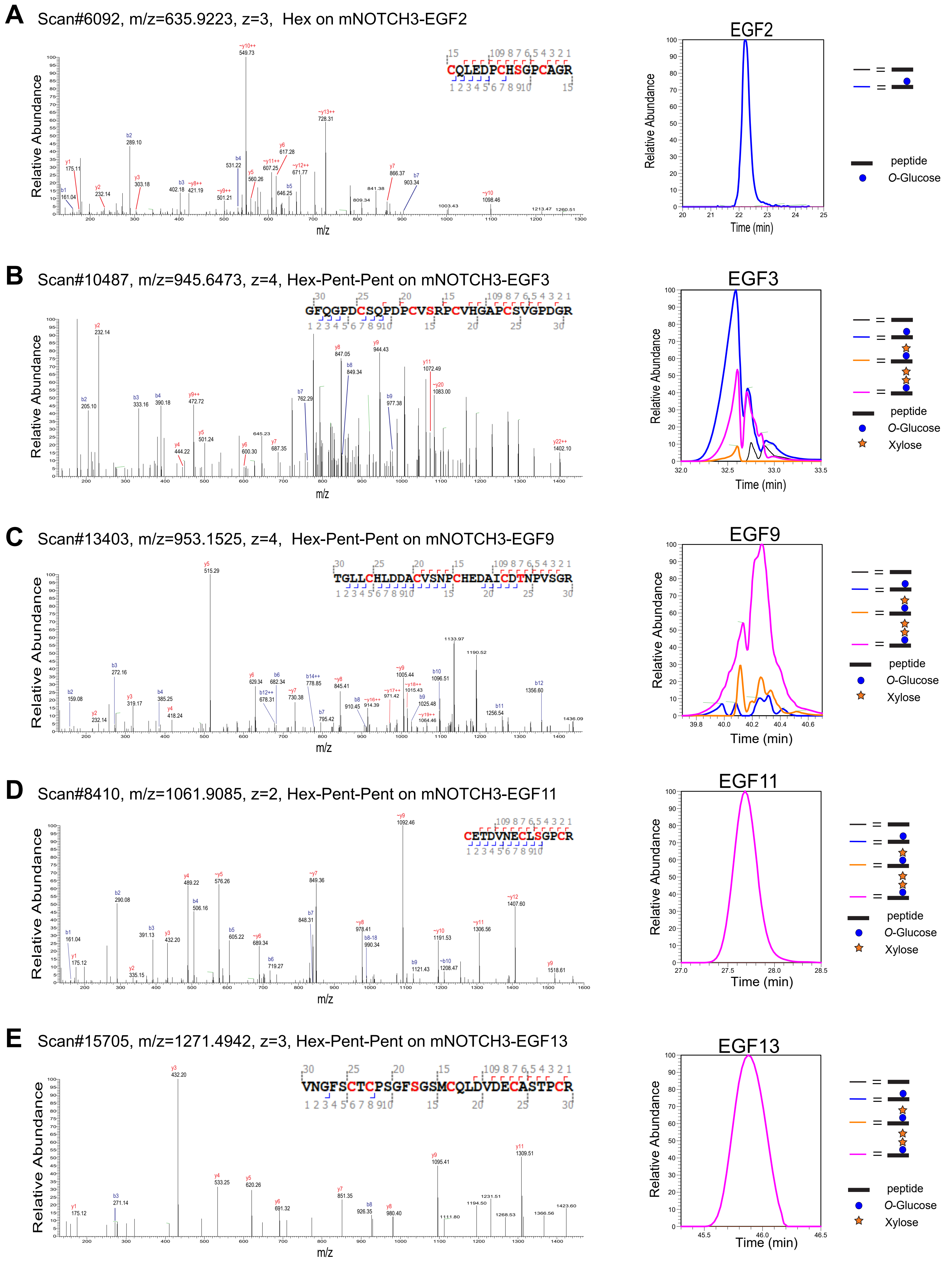
A
Scan#6092, m/z=635.9223, z=3, Hex on mNOTCH3-EGF2
CQLEDPCHSGPCAGR
Relative Abundance
m/z
EGF2
Time (min)
peptide
O-Glucose
B
Scan#10487, m/z=945.6473, z=4, Hex-Pent-Pent on mNOTCH3-EGF3
GFQGPDCSQPDPCVSRPCVHGAPCSVGPDGR
Relative Abundance
m/z
EGF3
Time (min)
peptide
O-Glucose
Xylose
C
Scan#13403, m/z=953.1525, z=4, Hex-Pent-Pent on mNOTCH3-EGF9
TGLLCHLDDACVSNPCHEDAICDTNPVSGR
Relative Abundance
m/z
EGF9
Time (min)
peptide
O-Glucose
Xylose
D
Scan#8410, m/z=1061.9085, z=2, Hex-Pent-Pent on mNOTCH3-EGF11
CETDVNECLSGPCR
Relative Abundance
m/z
EGF11
Time (min)
peptide
O-Glucose
Xylose
E
Scan#15705, m/z=1271.4942, z=3, Hex-Pent-Pent on mNOTCH3-EGF13
VNGFSCTCPSGFSGSMCQLDVDECASTPCR
Relative Abundance
m/z
EGF13
Time (min)
peptide
O-Glucose
Xylose

F Scan#14584, m/z=974.8969, z=4, Hex-Pent-Pent and HexNAc on mNOTCH3-EGF15

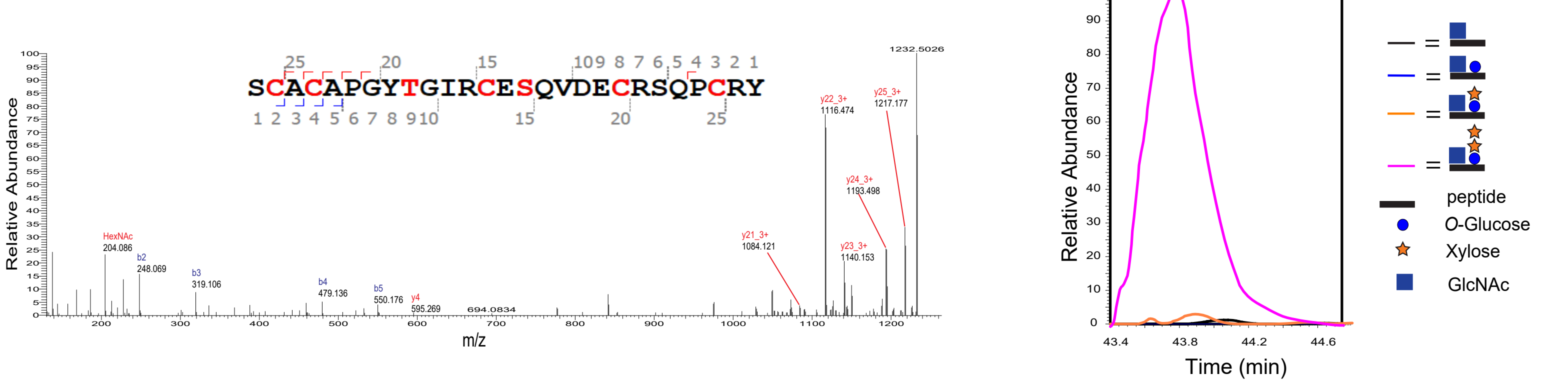

G Scan#18407, m/z=1248.5051, z=3, Hex-Pent-Pent and HexNAc on mNOTCH3-EGF16

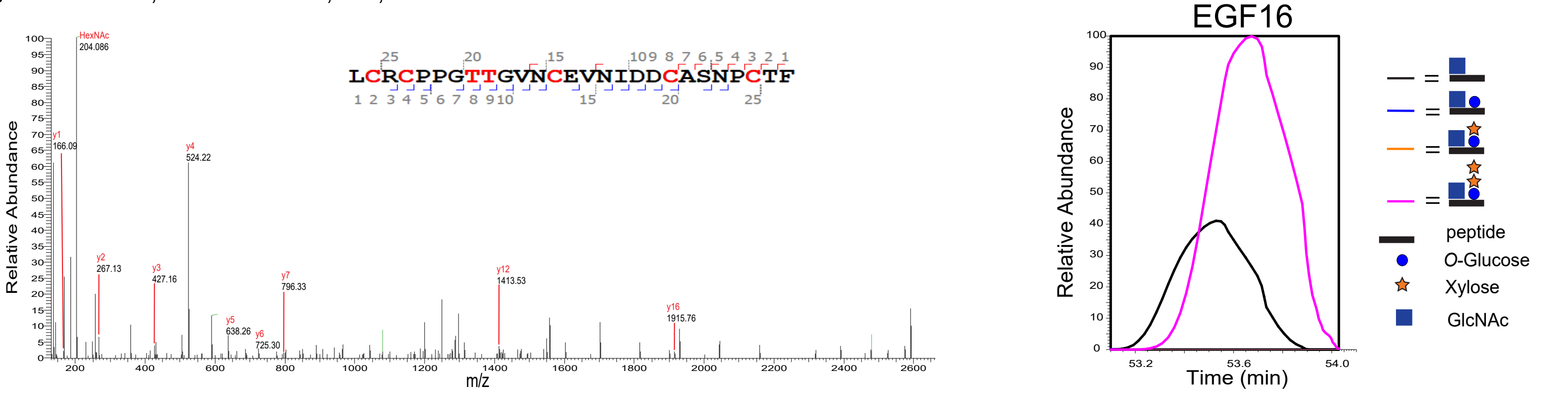

H Scan#17169, m/z=1581.8235, z=6, Hex-Pent-Pent and Fucose-HexNAc-Hex on mNOTCH3-EGF17

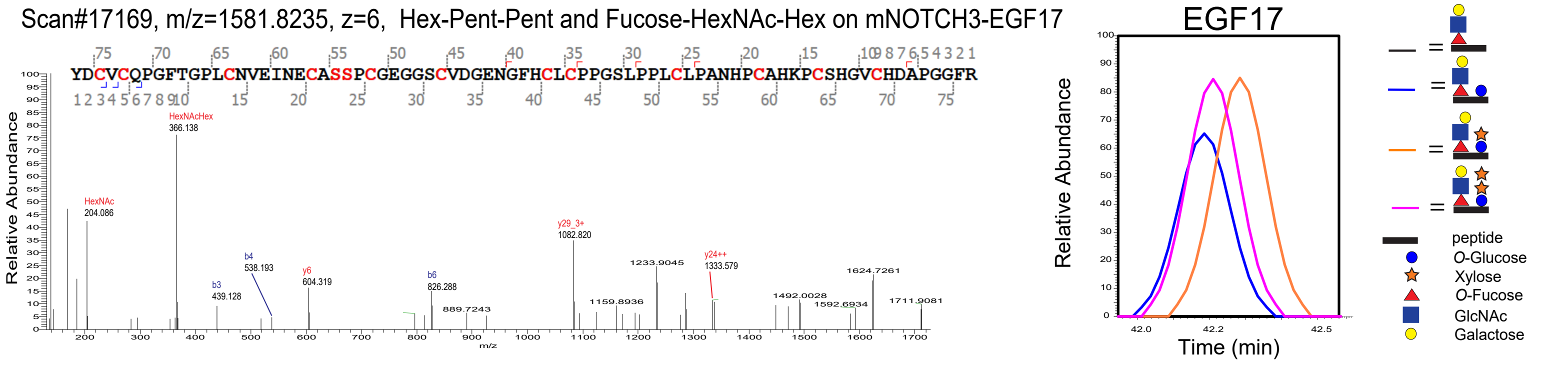

I Scan#12301, m/z=1230.4945, z=3, Hex-Pent-Pent and Fucose on mNOTCH3-EGF19

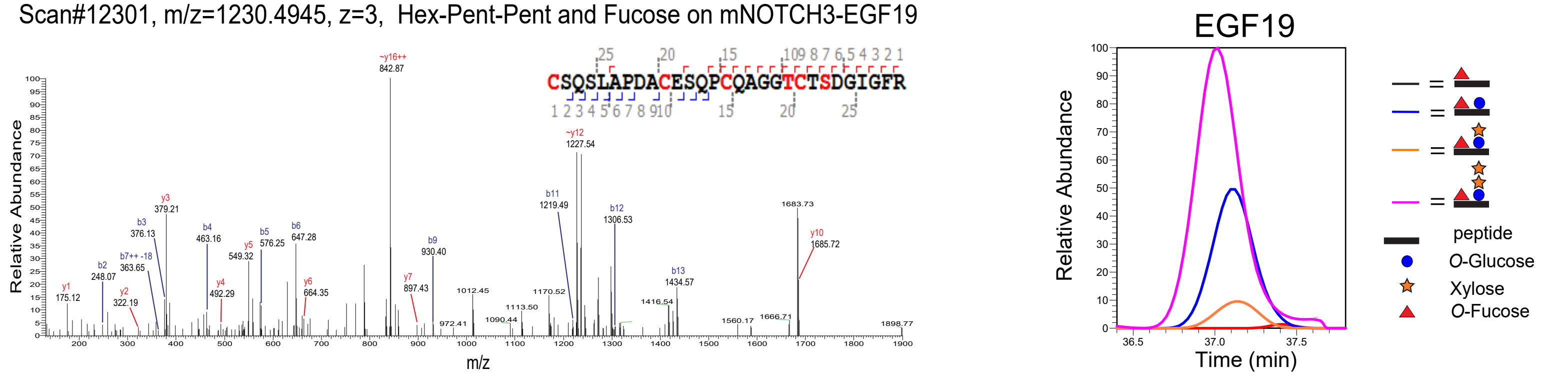

J Scan#13640, m/z=1018.4184, z=4, Hex-Pent-Pent and Fucose on mNOTCH3-EGF23

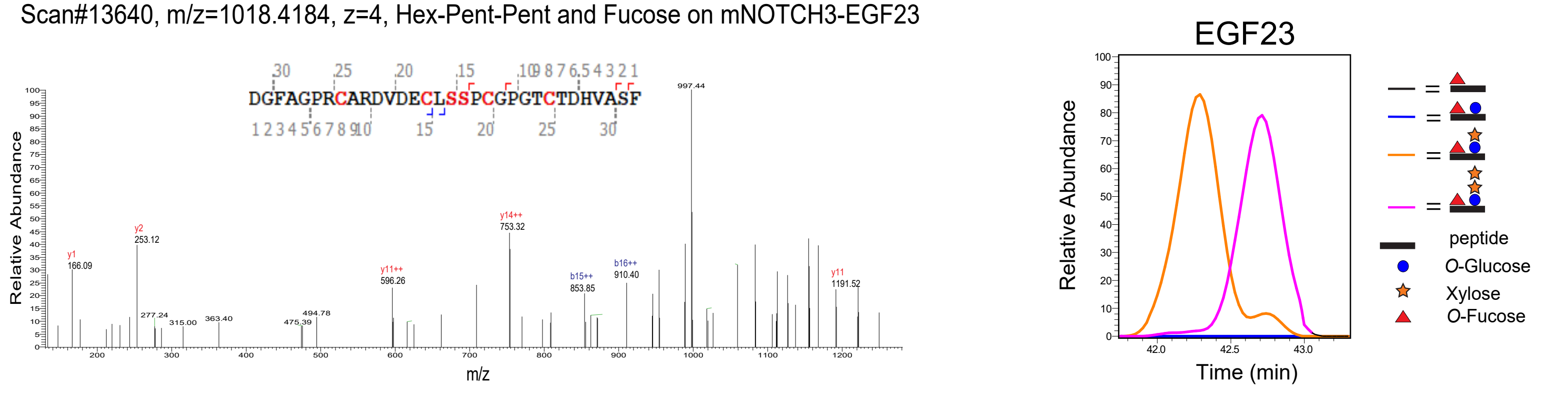

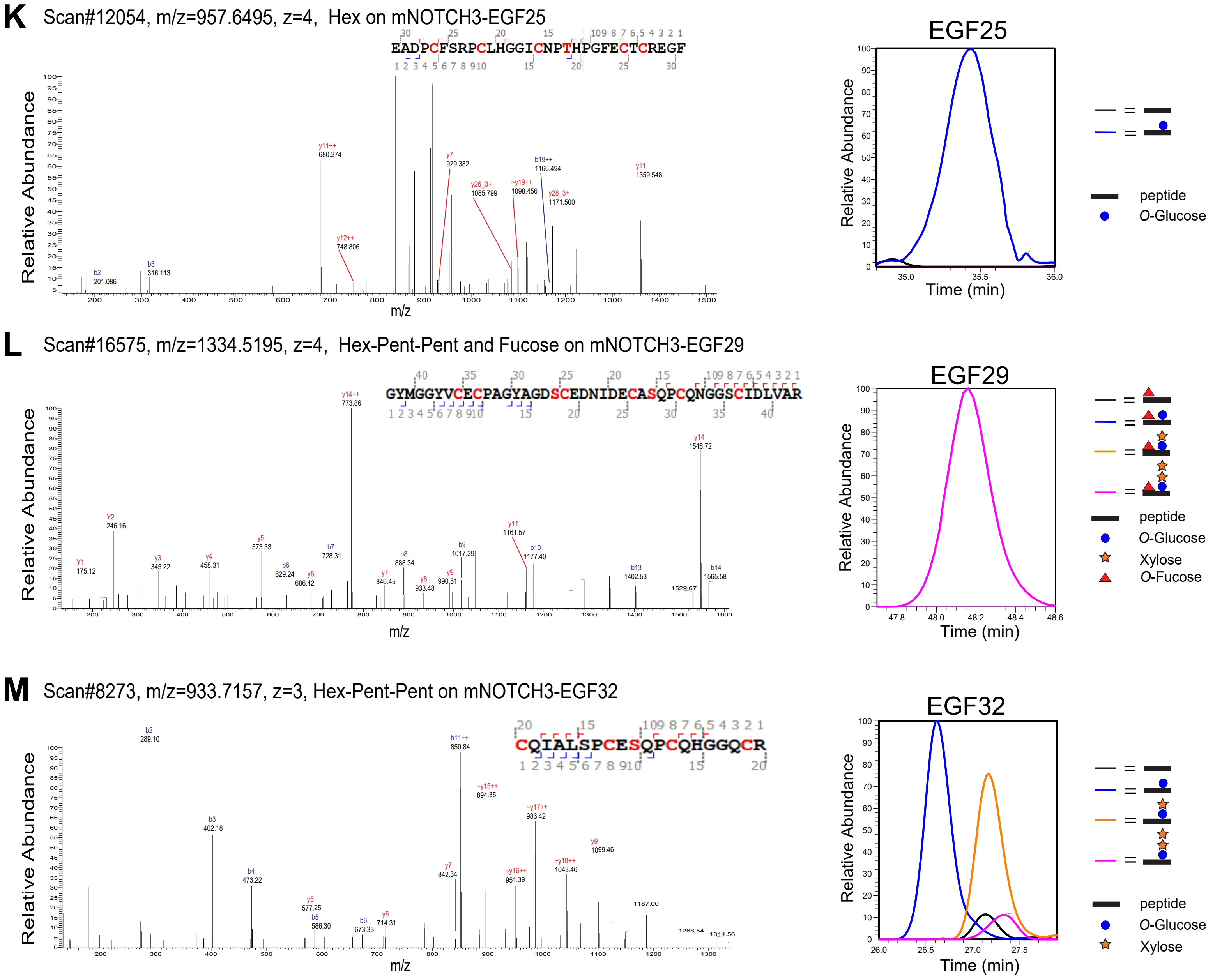
K
Scan#12054, m/z=957.6495, z=4, Hex on mNOTCH3-EGF25
EADPCFSRPCLHGGICNPTHPGFECTCREGF
Relative Abundance
m/z
y11++ 680.274
y12++ 748.806
y7 929.382
y26_3+ 1085.799
~y19++ 1098.456
b19++ 1166.494
y28_3+ 1171.500
y11 1359.548
b2 201.086
b3 316.113
EGF25
Time (min)
peptide
O-Glucose
L
Scan#16575, m/z=1334.5195, z=4, Hex-Pent-Pent and Fucose on mNOTCH3-EGF29
GYMGGYVCECPAGYAGDSCEDNIDECASQPCQNGGSCIDLVAR
Relative Abundance
m/z
y14++ 773.86
y14 1546.72
Y2 246.16
Y1 175.12
y3 345.22
y4 458.31
y5 573.33
b6 629.24
y6 686.42
b7 728.31
y7 846.45
b8 888.34
y8 933.48
y9 990.51
b9 1017.39
y11 1161.57
b10 1177.40
b13 1402.53
1529.67
b14 1565.58
EGF29
Time (min)
peptide
O-Glucose
Xylose
O-Fucose
M
Scan#8273, m/z=933.7157, z=3, Hex-Pent-Pent on mNOTCH3-EGF32
CQIALSPCESQPCQHGGQCR
Relative Abundance
m/z
b2 289.10
b3 402.18
b4 473.22
y5 577.25
b5 586.30
b6 673.33
y6 714.31
y7 842.34
b11++ 850.84
~y15++ 894.35
~y16++ 951.39
~y17++ 986.42
~y18++ 1043.46
y9 1099.46
1187.00
1268.54
1314.56
EGF32
Time (min)
peptide
O-Glucose
Xylose
