# Supplementary material for: The glycosyltransferase POGLUT1 regulates muscle stem cell development and maintenance in mice
Source: PLoS Genet. 2025 Aug 18;21(8):e1011806. doi: 10.1371/journal.pgen.1011806 (PMC12373270; doi:10.1371/journal.pgen.1011806)
Supplement: Attachment — Submitted filename: 152989_1_rebuttal_3373050_svncz5.pdf [file pgen.1011806.s007.pdf]

Full Revision

Manuscript number: RC-2024-02806R

Corresponding author(s): Hamed Jafar-Nejad, Carmen Paradas

1. General Statements [optional]

This section is optional. Insert here any general statements you wish to make about the goal of the study or about the reviews.

2. Point-by-point description of the revisions

Reviewer #1 (Evidence, reproducibility and clarity (Required)):

The manuscript by Cho et al uses conditional and inducible conditional mouse models to characterize the function of protein O-glucosyltransferase 1 (POGLUT1), known to cause a type of Limb Girdle Muscular Dystrophy (LGMD-R21), in skeletal muscle satellite cells, differentiation and regeneration. The Authors find that conditional deletion of POGLUT1 in the myogenic progenitors leads to postnatal muscle defects and lethality by postnatal day 30 or so. Muscle progenitors lacking POGLUT1 undergo reduced proliferation and accelerated differentiation, possibly leading to impairment in muscle regeneration. This is supported by an inducible conditional deletion of POGLUT1 in adult satellite cells. Finally, in vitro experiments suggest that POGLUT1 is required for NOTCH pathway activation in myogenic cells and that POGLUT1 could potentially glycosylate specific residues in NOTCH3.

- We thank the reviewer for the concise summary of the major findings of our manuscript and for her/his comments, which have helped us improve the manuscript.

Major comments

1. What is the control used in Figure 1B and other panels? The genotype should be clearly specified instead of saying controls, in all the figures. It will be easier to interpret the data if densitometry of the western blots is provided, normalized to GAPDH levels. Also, is there some POGLUT1 protein remaining in the satellite cells in the cKOs? Data should be shown for an adult time point also (P30 or later), in addition to P0 and P4, to see whether Poglut1 levels are reduced in adult stages in the muscle and satellite cells in the cKOs.

- As described in the Methods section of the original submission, for all experiments involving *Poglut1-cKO*, siblings without *Poglut1* deletion (*Poglut1*$^{+/+}$, *Poglut1*$^{flox/flox}$, or *Pax7*^{Cre}; *Poglut1*$^{+/+}$) were used as controls. To address the reviewer's comment, we added this information to the legend of all figures showing *Poglut1-cKO* data.
- In the revised version, the densitometry is provided for all western blot images (Figures 1B and 7A). We added western blot with anti-POGLUT1 on whole muscle extract from P21 animals (Figure 1B), which is the latest stage at which we consistently obtain *Poglut1-cKO* mice. PAX7 staining and injury studies indicate that at P21 there is a significant reduction in the number of satellite cells. Therefore, isolation of sufficient satellite cells from later stages is not feasible.

2. In Figure 1C, can the tibia weight be normalized to total body weight instead of tibia length and analyzed? Similarly, can the grip strength measurements in Figure 1G be normalized to total body weight and represented? Grip strength measurements in neonates is tricky; the Authors should clearly explain how this was done. The NMJ defects can be characterized better, especially since the Authors discuss about Agrin in detail.

- Normalizing tibialis anterior muscle weight to tibia bone length is commonly used and is especially useful when assessing muscle-specific growth or atrophy, as it provides a standardized measure to compare between different mice. To address the reviewer's comment, in the revised manuscript we have provided the tibialis anterior weight (not normalized), tibia length, and then the normalized TA weight (which was also shown in the first submission). Before normalization, the TA weight in cKO showed ~ 56% reduction compared to control (on average). The tibia length did not show a statistically significant difference between cKO and sibling control. After normalization to tibia length, the TA muscle weight still showed a substantial (55%) reduction in cKO mice compared to controls. These data indicate that a reduction in muscle size is the major if not the sole reason for the reduction observed in cKO body size.
- We reanalyzed the grip strength based on the reviewer's comment and found that when normalized to body weight, the grip strength is not significantly different between cKO and control animals (please see the following figure for the reviewer). Accordingly, we have modified the Results section to avoid any direct suggestion that the muscle function itself is impaired in the cKO animals. Specifically, instead of writing "… suggesting that the muscle function is impaired in the mutant mice" we wrote " … suggesting reduced muscle strength in the mutant mice". Moreover, at the end of this paragraph, instead of referring to "a severe muscle weakness" we just describe the phenotype as "muscle weakness". Our goal from this paragraph is to suggest that since the animals have much smaller muscles, they are weaker and cannot eat properly; that's why switching to soft food somewhat improves their survival. The observed NMJ defects suggest that there might be functional muscle defects as well.

However, to draw conclusions about the muscle function we will need additional experiments, which we believe are beyond the scope of the current manuscript. If the reviewer believes the normalized grip test should be added to the manuscript, we will be happy to do so as a supplementary figure.

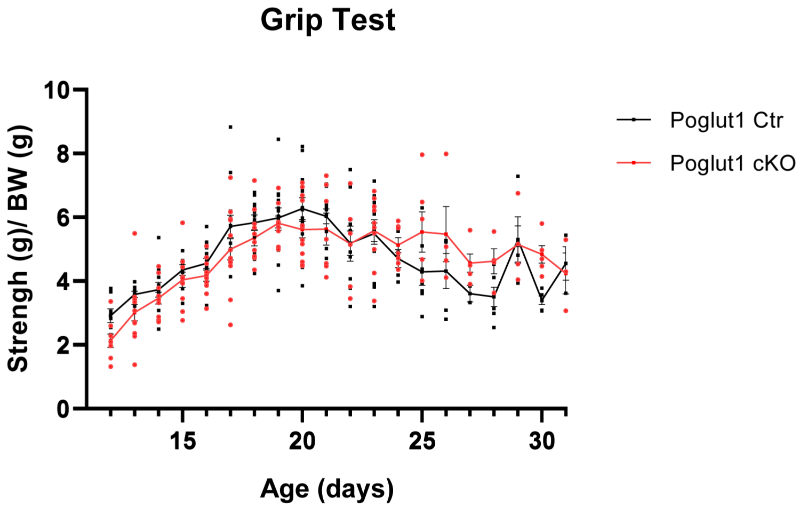

- As stated in the original manuscript, grip test was done starting at P12. Following the Boston University Guideline (https://www.bu.edu/research/ethics-compliance/animal-subjects/animal-care/anesthesia/anesthesia-and-analgesia-neonatal-mice-and-rats-iacuc/; recommended by Jacksons Laboratory when working with neonatal mice), we consider P10 to be the end of the neonatal period in mice. This is also supported by the majority of the papers in literature. For example, in a paper entitled "Quantitative analysis of neonatal skeletal muscle functional improvement in the mouse" (PMID 18310108), the authors show that by P28, most anatomical and gene expression parameters of mouse skeletal muscle are close to adults, and that by P21, these parameters are closer to P28 than the early postnatal mice. Also, they show that the expression of neonatal myosin heavy chain is dramatically reduced between P7 and P14, with another 3-4-fold reduction between P14 and P21. Therefore, for muscle development, similar to many other contexts studied in mice, neonatal period ends between P7 and P14 (most likely P8–P10). Therefore, the grip tests have not been performed on neonatal mice. In the revised manuscript, we have clarified this issue in the Methods section.
- The NMJ defects were not the main focus of the manuscript and were performed as a standard characterization of this new mouse model. We agree that additional experiments can be performed to better characterize the NMJ defects in this model, but we believe those experiments are beyond the scope of the current manuscript. Also, given the new data and

text added to the revised manuscript, we removed the section related to NMJ and agrin from the Discussion to reduce the manuscript length.

3. What are the small myofibers seen at the corners of the larger myofibers at P21 in the cKOs in Figure 2E? What is the point that the Authors want to conclude from Figure 2C and D? Clearly, if there are fewer satellite cells, Pax7 transcript levels will decrease. In Figure 2D, how are the satellite cell samples normalized between control and cKOs; did they start with equal number of satellite cells or equal amount of satellite cell RNA between control and cKOs? In Figure 2F, representative images for cKOs should be shown? The conclusion from Figure 2F-G is unclear. Since the Authors claim that the weak laminin staining is resolved in the cKOs by P21, why is α-dystroglycan hypoglycosylation seen in the P21 muscle?

- The structures to which the reviewer is referring are blood vessels. As shown in the following figure generated for the reviewer, staining the muscle sections from control and cKO mice using the endothelial marker CD31 confirms the vascular nature of those structures.

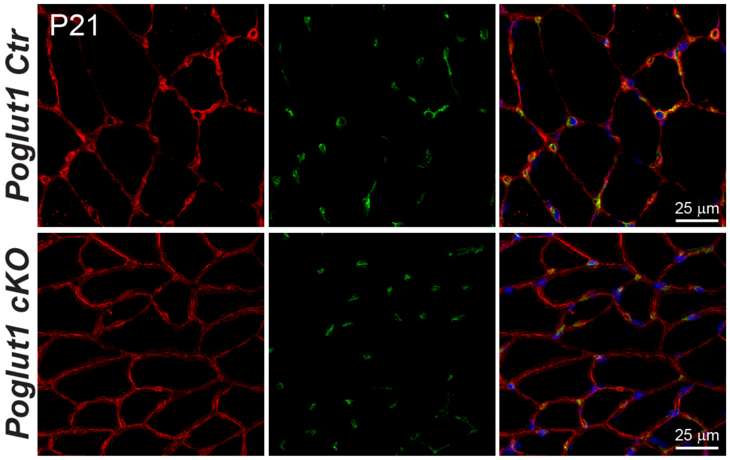

- The decrease in PAX7^{+} cells was shown based on antibody staining, but this does not necessarily mean that *Pax7* mRNA is similarly reduced. Figure 2C and 2D show that loss of *Poglut1* in these cells affects *Pax7* expression at the mRNA level. We started with equal amounts of RNA for Figure 2D and have added additional text to describe RNA isolation and RT-PCR in the Methods section of the revised manuscript.
- Representative images for cKOs are added to the revised Figure 2, as suggested.
- The conclusion from 2F-G is that a statistically significant reduction is specifically observed in those PAX7^{+} cells which co-express M-cadherin, which is another well-established satellite cell marker. The data also indicate that some PAX7^{+} cells persist in the mutant muscle, highlighting the importance of examining the ability

of mutant muscle to repair muscle injury, as shown in Figure 4.

- The glycans on α-dystroglycan mediate its binding to laminin and other extracellular matrix proteins, but laminin expression level is not thought to regulate the degree of α-dystroglycan glycosylation. In other words, laminin staining intensity and the degree of α-dystroglycan are not causally related to each other. What we observed in P21 animals mirrors what we have reported for adult patient biopsies: normal level of extracellular matrix protein collagen VI associated with α-dystroglycan hypoglycosylation. It is worth mentioning that based on our previous report using primary LGMD-R21 patient myoblasts and C2C12 cells treated with a Notch inhibitor, reducing Notch signaling leads to α-dystroglycan hypoglycosylation, likely by altering the differentiation dynamics of the myoblasts (PMID: 27807076).

4. Representative images should be shown as examples for all time points for both genotypes in Figure 3A. Figure 3H should be represented with statistically significant differences marked clearly. Have the Authors checked whether cell death contributes to the decrease in cultured satellite cells and Pax7+ cells in the cKOs in Figure 3F, G? Are the differences between the control and cKOs in fusion index and Myogenin expression (Figure 3J, K) statistically significant?

- Representative images are added in a new supplementary figure (Figure S3A) as requested by the reviewer.
- Thanks for pointing out the lack of statistics in Figure 3H. To address this point and a comment by reviewer 2, we removed those quantifications from the manuscript. Instead, we performed PAX7/MYOD/Ki67 co-staining on cultured cells and quantified the percentage of each cell state based on the expression of these three markers as a more accurate measure of quiescent versus cycling satellite cells, as well as progenitors and precursor cells. The revised quantification and statistical analysis is presented in the revised Figure 3H. This quantification is represented similarly to the data shown in Gattazzo et al 2020.
- Thanks for bringing up the issue of cell death. To address this point, we performed two rounds of anti-Caspase 3 staining on a limited number of myogenic progenitors that we were able to isolate from cKO and control animals during the revisions. Unfortunately, the stainings did not work. To ensure that we do not rule out the possibility of apoptosis without experimental data, we added the following sentence to the Results section: "These data suggest reduced proliferation of myogenic cells upon loss of *Poglut1,* although we cannot exclude that cell death also contributes to the reduced cell number."
- Thanks for pointing out the lack of statistics for these data. We have added additional independent samples to the data presented in 3J, K and found that the differences between the control and cKOs in fusion index and Myogenin expression are indeed statistically significant.

5. Figure 4 has multiple problems in my opinion. First, P21 is too early a time point to be talking about regeneration, since satellite cells are just transitioning to become quiescent cells at this time (described in Lepper et al, Nature 2009). It is difficult to distinguish the regenerative and neonatal developmental roles of satellite cells in the experiment that the Authors have carried out. Another issue is that the Authors show a decrease in satellite cells in the cKOs; satellite cell function is clearly known to be required for regeneration. Therefore, there is not much novelty that the cKOs exhibit poor regenerative capability. The data shown in Figure 4B-E is more or less a repetition of what has been shown in Figures 2 and 3.

- After carefully reviewing publications including the one suggested by the reviewer, we found that multiple studies have performed muscle injury experiments at P21 or even younger ages to study regeneration capabilities. Also, please consider that many of our mutant mice are lethal around weaning age which was another reason for selecting P21 as our injury timepoint. To better explain the logic for this choice, we have added the following sentences to the revised manuscript (new text is underlined: "To address this question, we induced muscle injury in mutant and control mice by injecting cardiotoxin (CTX) into TA muscles at P21 (Fig. 4A), an age at which we consistently obtain *Poglut1*-cKO animals without any dietary changes (Fig. 1E). Importantly, in WT C57BL/6 mice, 51% of PAX7^{+} cells are reported to be in the quiescent state at P21 (Gattazzo et al., 2020) and the TA muscles of P21 mice exhibit a robust regenerative response to cardiotoxin-induced injury (Lepper et al., 2009)."
- While the reduction in satellite cells (SCs) in cKOs suggests impaired regeneration, it was essential to confirm this experimentally, as remaining SCs could still compensate. Therefore, it was necessary to assess whether the residual satellite cells could proliferate and contribute to muscle repair. Our results demonstrate that our mutant mice fail to repair muscle, providing evidence that the reduction in PAX7^{+} cells in *Poglut1-cKO* mice is functionally significant. The goal of these experiments was not to increase novelty; it was to increase experimental rigor and reproducibility so that we don't draw functional conclusions merely based on tissue staining.
- While Figures 2 and 3 examine muscle sections and myoblast cultures, Figure 4B-E presents isolated fibers in an ex vivo culture system. This approach allows us to assess satellite cell activation and proliferation in a different injury model. Figure 4 lets us confirm that the proliferation defect observed in the cKO is consistent across multiple experimental conditions. Additionally, we utilized two forms of muscle injury—in vivo and ex vivo—to comprehensively evaluate the function of PAX7^{+} cells in WT versus mutant muscles. The ex vivo findings from EDL fibers align with our cell culture experiments in Figure 3, further

supporting the observed defect in muscle repair. We believe this approach will increase the likelihood of obtaining reproducible data.

6. In Figure 5C, D, have the Authors checked the Pax7+ cell numbers between the controls and the i-cKOs? Is the difference seen in 1X TAM due to preexisting reduction in satellite cells in the i-cKOs? What is the explanation by the Authors for the large number of Tomato+ fibers seen in the 2X TAM and 3X TAM uninjured muscle (Figure 3C, E)? Figure 5E is difficult to comprehend and should be represented in a clearer manner.

- New PAX7 staining shows that most of the TOM^{+} cells (93% in *i-cKO*, 97% in control) co-express PAX7 (revised Figure 6D and 6E). If preexisting meant "before tamoxifen injection", a preexisting reduction in satellite cells should be highly unlikely, as the Pax7-Cre-ERT2 line has been used by multiple groups to study adult satellite cells, and we have not observed any abnormalities in the Poglut1[flox/flox] strain. Moreover, our control animals harbor both Pax7-Cre-ERT2 and the tdTomato transgenes. The observed difference in 1xTAM most likely reflects spontaneous activation and fusion of these cells following loss of *Poglut1*, as evidenced by the enhanced appearance of Tomato+ fibers in *i-cKO* muscles compared to control muscles. At 1xTAM, we can observe a modest increase in Tomato^{+} myofibers. The reduction becomes more pronounced with repeated tamoxifen, consistent with progressive differentiation of the satellite cell pool and their fusion with myofibers.
- For Figure 5E (now Figure 6C), we made some changes to make the graph clearer and added a sentence to the figure legend to make it easier to follow.

7. Densitometry for Figure 6A should be included, since N1 ICD levels seem quite variable in the controls also. POGLUT1 expression and shRNA knockdown efficiency should be shown in Figure 6B. What is the correlation between the NOTCH3 glycosylation with reduced NOTCH pathway activation and satellite cell function? This should be clearly discussed. Why was the NOTCH3 glycosylation assay done in HEK293 cells?

- In the revised version, we have included densitometry for Figure 6A (now Figure 7A).
- In the revised version, we show qRT-PCR data indicating that C2C12 cells stably expressing *Poglut1* shRNA show ~ 75% decrease in *Poglut1* mRNA levels compared to control cells (now Figure 7B), in agreement with our original report of these cells (PMID 21490058). We also performed qRT-PCR for *Pax7* and found a significant reduction in *Pax7* mRNA levels in *Poglut1*-knockdown C2C12 cells, further supporting their usage in these signaling assays.
- As discussed in detail in the Results and Discussion sections of the original submission, reports from our lab and others have provided strong evidence that glycosylation of NOTCH1 and NOTCH2 by POGLUT1 promotes signaling mediated by these receptors.

Since it was not clear whether this phenomenon is ligand-specific, in the current manuscript we showed that both DLL1-madiated and JAG1-mediated signaling by NOTCH1 and NOTCH2 require the expression of POGLUT1 in the signal-receiving cells. Since NOTCH3 also plays a key role in muscle stem cell biology (albeit a not so well characterized one), we showed in the current manuscript that reducing POGLUT1 in the NOTCH3-expressing cell also leads to a significant reduction in signaling mediated by this receptor. Moreover, we found that NOTCH3 is broadly glycosylated by POGLUT1. In fact, during the revision, we succeeded in confirming four additional POGLUT1 target sites on the mouse NOTCH3 protein (added to the revised Figure 7D and Supplementary Figure S4). These data suggest that similar to NOTCH1 and NOTCH2, POGLUT1-medaited O-glucosylation of NOTCH3 is required for its signaling. To address the reviewer's comment, in the revised manuscript, we have added the following phrase to the Discussion to address the reviewer's comment: "…, strongly suggesting that *O*-glucosylation of NOTCH3 by POGLUT1 promotes its ligand-mediated activation."

- HEK293 cells express robust levels of POGLUT1 and have been used in previous studies for the characterization of POGLUT1-mediated glycosylation of its target proteins. Analysis of overexpressed target proteins in this cell line is used to establish assay conditions and glycopeptide identification parameters and thereby sets the stage to analyze glycosylation of endogenous protein (in this case, mouse NOTCH3) in specific cell types. We note that in cases examined so far, the glycosylation data obtained from overexpressed proteins in HEK293 or other commonly used mammalian cell lines recapitulate the glycosylation of endogenously expressed POGLUT1 targets very well (both in cell culture and in vivo; for example see PMID 27268051).

Reviewer #1 (Significance (Required)):

Based on my expertise as a muscle and stem cell biologist, the manuscript is not clearly thought through, with not many novel inferences that one can draw from the data provided. While the manuscript could be informative to muscle biologists and stem cell investigators, several additional experiments are required to better characterize the phenotypes and provide meaningful conclusions from the study. The role of POGLUT1 in the muscle could be of great interest, especially in light of its role in LGMD-R21, as described by some of the Authors previously. Several pieces of data provided in the current manuscript are disjointed, with few connecting links, such as the NMJ characterization, the NOTCH glycosylation data and the regeneration experiments done on P21 neonates. Better quantitation of data is required as well, as detailed below. Overall, the manuscript may be

revised to address the specific comments and reconsidered at a suitable journal.

Response: We hope that our explanations, additional experiments, and changes to the text have helped address the concerns raised by the reviewer.

Reviewer #2 (Evidence, reproducibility and clarity (Required)):

Poglut1 mutations have been identified in an autosomal recessive form of muscular dystrophy. Poglut1 encodes an O-glucosyltransferase that modifies Notch receptors and ligands but also has other substrates. In mice, null mutations in Poglut1 are embryonic lethal, which has previously precluded the analysis of Poglut1's role in muscle development and regeneration using murine models. To address this limitation, the authors generated a conditional Poglut1 allele and introduced mutations using Pax7Cre or Pax7CreERT2. They characterize the phenotypic consequences of these mutations and further demonstrate that signaling through the Notch1, Notch2, and Notch3 receptors requires Poglut1 using the C2C12 cell culture model.

Some aspects of the manuscript's description of muscle stem cell behavior and regeneration are not fully up to date. These points should be addressed before publication.

Specific points

1) Evidence Supporting Reduced Notch Signaling as the Cause of the Phenotype
The comparison of muscle phenotypes observed in other Notch signaling mutations strongly supports the hypothesis that the phenotype is due to reduced Notch signaling. Relevant studies, such as those addressing development (PMID: 17194759, PMID: 17360543) and those focusing on adult muscle and regeneration (PMID: 21989910, PMID: 22069237, PMID: 22045613, PMID: 30862660) should be cited and discussed in the manuscript. Including these references will strengthen the argument and provide a broader context for the findings.

- We thank the reviewer for this comment. To address this point and a similar comment made by reviewer 3, we have modified the Introduction and Discussion in the revised manuscript. In the Introduction, we have added the following sentences and have cited all 6 references

listed by the reviewer: “Loss of function studies for key components of the Notch signaling pathway including *Rbpj* and *Dll1* indicated that disruption of this pathway during muscle development leads to premature differentiation and depletion of muscle progenitor cells (Schuster-Gossler et al., 2007; Vasyutina et al., 2007). In addition, multiple studies have shown that Notch signaling is required in adult mice to prevent the spontaneous or premature differentiation of satellite cells and to maintain a muscle stem cell pool capable of repairing muscle damage (Bjornson et al., 2012; Fukada et al., 2011; Lahmann et al., 2019; Mourikis et al., 2012).” In the third paragraph of the Discussion, we have highlighted the similarities between *Poglut1-cKO* and *i-cKO* phenotypes and the phenotypes observed in “animals with germline or conditional loss of various Notch pathway components (Bjornson et al., 2012; Fukada et al., 2011; Lahmann et al., 2019; Mourikis et al., 2012; Schuster-Gossler et al., 2007; Vasyutina et al., 2007)”.

2) Figure 1: NMJ Deficits - Pre- or Postsynaptic?
The authors describe the mutant synaptic vesicles as showing a significantly reduced evoked neurotransmitter release (quantal content) compared to controls. This phrasing raises the question: are motor neurons mutated in these animals? It should be clarified why the synaptic vesicles are referred to as "mutant." To my knowledge, Pax7Cre does not recombine in motor neurons, and this discrepancy needs to be addressed. The text should be rephrased to accurately reflect the origins of the observed deficits.

- We are not aware of any reports on recombination in motor neurons by the Pax-Cre line used in our study and had implied this in the initial submission in the following sentence in Discussion: “Therefore, even if agrin is indeed glycosylated by POGLUT1, loss of *Poglut1* with *Pax7-Cre* is not expected to affect the glycosylation of agrin expressed by motoneurons.” [please note that given the new data and text added to the revised manuscript, we removed the paragraph related to NMJ and agrin from the Discussion to reduce the manuscript length.] We agree that referring to synaptic vesicles as “mutant” can be misleading and have revised “mutant synaptic vesicles” to “in the NMJs of *Poglut1-cKO* LAL muscles”. To address the discrepancy, we have added the following sentence to the Results section after describing a reduction in quantal content: “Since the *Pax7-Cre* strain used in our study is not reported to induce recombination in motor neurons (Murdoch et al., 2012), this presynaptic NMJ defect might be secondary to defects in postsynaptic NMJ abnormalities.”

3) Quantification of Myofibers with Internal Nuclei
The statement, "We first quantified the ratio of myofibers with internal nuclei, which is an indication for recent fusion of myoblasts to myofibers," is not entirely accurate. Recent

studies, such as PMID: 38569550, provide a more nuanced explanation of this phenomenon. The manuscript should reference this study and update the description to ensure it accurately reflects the current understanding of myofiber internal nuclei as markers of muscle pathology or regeneration.

- We thank the reviewer for bringing this paper to our attention. In the revised manuscript, we have changed the above sentence to make it aligned with the observations of the paper mentioned by the reviewer: "We first quantified the ratio of myofibers with internal nuclei, recently reported to specifically result from the fusion of embryonic myogenic cells during limb myogenesis and also driven by myocyte-myocyte fusion in the first phase of postnatal muscle regeneration (Collins et al., 2024)." We also added the following sentence, which we believe makes our interpretation more accurate in light of Collins et al, 2024: "These data suggest enhanced differentiation of *Poglut1*-deficient progenitors into myocytes followed by continued myocyte-myocyte fusion and/or a delay in the peripheral migration of internal nuclei which normally occurs in the perinatal period (Collins et al., 2024)." This way, in the revised manuscript we do not link the presence of internal nuclei with recent fusion of myoblasts anymore. Finally, we also referred to Collins et al 2024 when describing the appearance of internal nuclei in injured muscles 5 days after injury (Figure 4A).

4) Figure 3H: Cell Identification in Culture

The use of PAX7+ MYOD− to identify quiescent cells in culture is not accurate. Instead, PAX7+ Ki67− should be used for this purpose.
Similarly, PAX7− MYOD+ does not reliably identify differentiating cells. Instead, staining for MYOG should be used to ensure accuracy.
The figure and accompanying text should be adjusted accordingly to reflect these updates.

- To address this issue and a comment by reviewer 1, we performed PAX7/MYOD/Ki67 co-staining on cultured cells and quantified the percentage of each cell state based on the expression of these three markers as a more accurate measure of quiescent versus cycling satellite cells, as well as progenitors and precursor cells. The revised quantification and statistical analysis is presented in the revised Figure 3H. This quantification is represented similarly to the data shown in Gattazzo et al 2020. Based on the reviewer's comment and the results of these quantification, we have modified the Results section as follows (new text is underlined): "These observations <u>indicate</u> that <u>loss of *Poglut1* impairs the ability of muscle stem cells to remain in a quiescent state and suggest that the</u> mutant myogenic progenitors <u>might undergo</u> premature differentiation." We then present the fusion index and myogenin staining data to conclude enhanced differentiation.

5) Description of Satellite Cell Quiescence

The statement, "About 2-3 weeks after birth, some of the PAX7+ cells generated by active proliferation of embryonic myogenic progenitors enter a quiescent state to generate adult satellite cells," is not entirely correct.
The description should be updated based on the findings in PMID: 32763161, which provide a more accurate account of the transition of PAX7+ cells to quiescence and their role in generating adult satellite cells.

- We thank the reviewer for bringing this paper to our attention. To address this issue and one of the concerns raised by Reviewer 1, we have made the following changes to the corresponding paragraph in the revised version: (A) We removed the statement highlighted by the reviewer. (B) We provided better justification for using P21 mice to assess muscle regeneration by quiescent satellite cells by adding the underlined sentences to this section: "To address this question, we induced muscle injury in mutant and control mice by injecting cardiotoxin (CTX) into TA muscles at P21 (Fig. 4A), an age at which we consistently obtain *Poglut1*-cKO animals without any dietary changes (Fig. 1E). Importantly, 51% of PAX7^{+} cells are reported to be in the quiescent state at P21 (Gattazzo et al., 2020) and the TA muscles of P21 mice exhibit a robust regenerative response to cardiotoxin-induced injury (Lepper et al., 2009)." We note that Gattazzo et al 2020 is PMID: 32763161, the paper with a more accurate account of satellite cell quiescence mentioned by the reviewer.

6) Figure 5: Loss of Quiescence in Satellite Cells

A hallmark phenotype of mutations in Notch signaling genesis the loss of quiescence in satellite cells when the mutation is introduced in the adult,.
The authors should include data on Ki67 and MyoD expression in PAX7+ cells of mice with the Poglut1 mutation introduced in the adult by an analysis of the uninjured muscle. This would provide insight into the maintenance of quiescence in the mutant satellite cells.

- Based on the reviewer's recommendation, we aimed to assess Ki67 and MyoD expression in PAX7^{+} cells of adult inducible-*cKO* (*i-cKO)* mice in the uninjured muscle. However, due to technical limitations including antibody species incompatibility and the number of available fluorescence channels, we were unable to perform simultaneous triple staining combined with tdTomato visualization on the same tissue sections. Instead, we utilized the Tomato reporter as a marker for PAX7^{+} cells, based on new data indicating that 93-97% of TOM^{+}

cells co-express PAX7 (Figure 6E). We performed Ki67 and MyoD double staining and quantified the percentage of Tomato$^+$ cells that expressed one or both of these markers. Our analysis showed that TOM$^+$/Ki67$^-$/MyoD$^-$ (quiescent) satellite cells were significantly reduced in the *i-cKO* mice compared to controls, while the proportions of single-positive cells (Ki67$^+$ or MyoD$^+$) were increased (Figure 6F-G). These findings are consistent with loss of quiescence and increased activation of satellite cells upon loss of POGLUT1 in the adult mice.

- During the revision, we performed additional experiments (not suggested by the reviewers) to better assess the role of *Poglut1* in self-renewal of satellite cells upon injury. First, in a new cohort of *i-cKO* and control animals, we performed two rounds of tamoxifen injections but skipped the first round of injury and only induced CTX injury after the second round of recombination. Interestingly, analysis of 14 dpi muscles from these animals showed a full repair in both control and *i-cKO* mice (shown in revised Figure 5B). Second, we inspected the repaired control and *i-cKO* muscle after one round of recombination + injury for the presence of single tdTomato$^+$ cells next to the repaired myofibers, which would represent the satellite cells formed after the repair. While single tdTomato$^+$ cells were readily seen in the repaired muscle in control mice, we did not see any such cells next to the repaired muscle from *i-cKO* animals (shown in the revised Fig 5C, n = 4 animals per condition). Together, these new data provide strong evidence that loss of *Poglut1* in adult muscle stem cells impairs their ability to return to quiescence and form new satellite cells upon repair of injured muscle.

7) Figure 6A: Assay of Cleaved NOTCH1 Intracellular Domain

The text describes that satellite cells isolated from Poglut1-cKO muscles showed a strong reduction in the level of cleaved (active) NOTCH1 intracellular domain compared to control cells.

It is unclear whether these satellite cells were freshly isolated and directly assayed or cultured before the assay. Please specify this in the result section.

- These satellite cells were freshly isolated from whole muscle, and the proteins were extracted directly from the isolated satellite cells without culturing them. This is now included in the Results section related to the revised Figure 7A.

8) Figure 6: Notch Receptor Overexpression in C2C12 Cells

The results section should explicitly explain that the Notch 1-3 receptors were overexpressed or transfected in the C2C12 cells. This detail is essential for understanding the experimental design.

- Thank you. The C2C12 cells were transfected with mouse NOTCH receptors 1, 2, or 3 to overexpress each receptor. This is now included in the Results section as suggested.

Reviewer #2 (Significance (Required)):

The manuscript by Cho et al. demonstrates that the muscular dystrophy phenotype associated with Poglut1 mutations is caused by a Notch signaling deficit in muscle stem cells. Although previous studies had suggested this connection, alternative mechanisms could not be excluded, as Poglut1 glycosylates a multitude of proteins. Overall, this is a thorough and careful analysis of Poglut1's role in muscle development and regeneration, providing valuable insights into the mechanisms underlying this rare muscle disease.

Response: We sincerely thank the reviewer for her/his positive assessment of our manuscript and for the constructive comments.

Reviewer #3 (Evidence, reproducibility and clarity (Required)):

POGLUT1 is a key enzyme essential for the glycosylation and signaling activity of Notch receptors in C2C12 muscle cells. Elegant work from multiple groups has demonstrated the essential role of Notch signaling in maintaining the quiescence of muscle stem cells known as satellite cells. For instance, genetic deletion of Rbpj in tamoxifen-inducible conditional knockout mouse models causes ectopic expression of MyoD, diminished Pax7 expression, and precocious differentiation and fusion of quiescent satellite cells without entering the cell cycle. This is accompanied by the complete loss of tissue regeneration following repetitive muscle injuries. Hypomorphic mutations of POGLUT1 cause limb-girdle muscular dystrophy (type R21), accompanied by a significant reduction of Notch signaling and a decrease in the number of satellite cells in patient muscles.

Despite the substantial body of evidence establishing the key function of the POGLUT1-Notch-Pax7/MyoD axis in satellite cells, the in vivo function of POGLUT1 in mice has not been studied. In the present study, Cho et al. carefully examined muscle development and regeneration using the POGLUT1 conditional knockout mouse model. The results, at both

phenotypic and cellular/molecular levels, align perfectly with the previously established working model of the POGLUT1-Notch-Pax7/MyoD axis. Overall, the experiment is well-designed and executed, and the data are generally of high quality supporting the conclusions. No specific experimental issues was identified.

Major suggestions include adding references in the discussion section: 1) studies of Rbpj cKO in mice (PMID: 22069237; 22045613), as genetic deletion of POGLUT1 using the same Pax7 driver showed almost identical phenotypes across all levels: phenotype, cellular fate changes, and gene expression changes; 2) studies of Notch-Rbpj targets (Fig. S1, PMID: 29795344), as this paper identified multiple genes encoding collagen V and VI as direct targets of Notch signaling in quiescent satellite cells. These findings are consistent with the authors' observations in Fig. 2e.

- We thank the reviewer for the suggestion to add these highly relevant papers to the manuscript. 1) To address this comment and the specific point #1 raised by reviewer 2, we have highlighted the similarities between *Poglut1-cKO* and *i-cKO* phenotypes and the phenotypes observed in "animals with germline or conditional loss of various Notch pathway components (Bjornson et al., 2012; Fukada et al., 2011; Lahmann et al., 2019; Mourikis et al., 2012; Schuster-Gossler et al., 2007; Vasyutina et al., 2007)" in the Discussion. Mourikis et al 2012 and Bjornson et al 2012 are the two papers that the reviewer has referred to. 2) To address this point, we have added the following sentences to the Discussion: "Notch signaling has been shown to directly activate the transcription of several genes encoding collagen V and VI in muscle stem/progenitor cells, and collagen V expressed by satellite cells plays a key role in the niche to maintain satellite cell quiescence (Baghdadi et al., 2018). Therefore, ECM abnormalities caused by reduced Notch signaling might contribute to the loss of satellite cell quiescence in *i-cKO* animals as well." Baghdadi et al, 2018 is PMID: 29795344, to which the reviewer has referred.

Minor suggestions:
Fig. 1B: "P0" is an awkward term. Does it refer to newborn day 1 or a near full-term embryo?

- By writing "P0", we are referring to the day the litters are born.

Fig. 1C: The y-axis label includes an extra space.

- Thanks. The extra space is removed in the revised figure.

Fig. 1D: Provide the N number for each genotype/diet group.

- The N number has been added (now Figure 1E).

Gene nomenclature: Use POGLUT1 exclusively for the human protein and Poglut1 for the mouse protein.

- According to the guidelines of the International Committee for Standardized Genetic Nomenclature for Mice, protein symbols for mice should use all uppercase letters. Therefore, while the gene symbols for mouse and human are different in terms of uppercase versus lowercase usage, the protein symbols for mouse and human are usually identical. Please see the following webpage: https://www.informatics.jax.org/mgihome/nomen/gene.shtml#ps (accessed January 18, 2025).

Reviewer #3 (Significance (Required)):

In the present study, Cho et al. carefully examined muscle development and regeneration using the POGLUT1 conditional knockout mouse model. The results, at both phenotypic and cellular/molecular levels, align perfectly with the known working model of the POGLUT1-Notch-Pax7/MyoD axis in satellite cells and muscle regeneration. Overall, the experiment is well-designed and executed, and the data are generally of high quality.

Response: We sincerely thank the reviewer for the positive evaluation of our manuscript as well as the helpful suggestions.
