# Supplementary material for: The glycosyltransferase POGLUT1 regulates muscle stem cell development and maintenance in mice
Source: PLoS Genet. 2025 Aug 18;21(8):e1011806. doi: 10.1371/journal.pgen.1011806 (PMC12373270; doi:10.1371/journal.pgen.1011806)
Supplement: Attachment — Submitted filename: R2- Response to reviewers.pdf [file pgen.1011806.s008.pdf]

Response to reviewer comments

Reviewer #1: The authors have addressed all my concerns. Congratulations!

Thank you!

Reviewer #2: The Authors have addressed or explained most of my queries. My only comments are the following:

We thank the reviewer for his constructive feedback.

1. The Authors choose to use controls which are siblings but are of mixed genotypes, which I am not sure of. How are the Authors confident that all control genotypes (Poglut1+/+, Poglut1flox/flox, or Pax7Cre; Poglut1+/+) are identical?

We selected these control genotypes for several reasons. First, all of the control animals used in our analysis do not have deletion of *Poglut1*. Including multiple control genotypes allows us to minimize potential genotype-specific bias that might arise from relying on a single control genotype, especially in experiments involving multiple genotypic combinations. Second, we used littermates as controls to ensure close matching with mutants in terms of maternal care, litter size, and developmental timing. Importantly, the control datapoints are generally consistent, without much overlap with the mutant datapoints. Based on these observations, we believe it is appropriate and even preferable to use these genotypes in combination as controls in this context compared to selecting one specific genotype.

2. How many biological replicates have been used for the P21 timepoint in Figure 1B and C? A minimum of 3 replicates are required for drawing conclusions from statistical tests.

For the Western blot image and quantification shown in Figure 1B and C, we used two biological replicates. We agree that three or more biological replicates are needed for statistical analysis. However, in this case, we are not making any statistical arguments. The data are shown to illustrate a trend consistent with other results in the manuscript.

3. The grip strength data normalized to body weight should be included in the manuscript.

We have now included the grip strength data normalized by body weight in Fig S1 and added the following sentence to the Results section to explain the data: "Of note, when normalized by body weight, grip strength did not differ significantly between *Poglut1-cKO* and control animals, suggesting that the observed reduction in absolute grip strength in mutants can be attributed to their reduced body weight (Supplementary Fig. S1A)".

Response to Journal Requirements:

1) We ask that a manuscript source file is provided at Revision. Please upload your manuscript file as a .doc, .docx, .rtf or .tex. If you are providing a .tex file, please upload it under the item type u2018LaTeX Source Fileu2019 and leave your .pdf version as the item type u2018Manuscriptu2019.

Done.

2) Please provide an Author Summary. This should appear in your manuscript between the Abstract (if applicable) and the Introduction, and should be 150-200 words long. The aim should be to make your findings accessible to a wide audience that includes both scientists and non-scientists. Sample summaries can be found on our website under Submission Guidelines:
https://journals.plos.org/plosgenetics/s/submission-guidelines#loc-parts-of-a-submission

Done.

3) We do not publish any copyright or trademark symbols that usually accompany proprietary names, eg ©, ®, or TM (e.g. next to drug or reagent names). Therefore please remove all instances of trademark/copyright symbols throughout the text, including:
- ® on page: 19
- TM on pages: 19, and 20.

Removed.

4) Please upload all main figures as separate Figure files in .tif or .eps format. For more information about how to convert and format your figure files please see our guidelines:
https://journals.plos.org/plosgenetics/s/figures

Figures were run through PACE and then uploaded as individual .tif files.

5) Some material included in your submission may be copyrighted. According to PLOSu2019s copyright policy, authors who use figures or other material (e.g., graphics, clipart, maps) from another author or copyright holder must demonstrate or obtain permission to publish this material under the Creative Commons Attribution 4.0 International (CC BY 4.0) License used by PLOS journals. Please closely review the details of PLOSu2019s copyright requirements here: PLOS Licenses and Copyright. If you need to request permissions from a copyright holder, you may use PLOS's Copyright Content Permission form.
Please respond directly to this email and provide any known details concerning your material's license terms and permissions required for reuse, even if you have not yet obtained copyright permissions or are unsure of your material's copyright compatibility.

Once you have responded and addressed all other outstanding technical requirements, you may resubmit your manuscript within Editorial Manager.
Potential Copyright Issues:
i) Please confirm (a) that you are the photographer of 1A, or (b) provide written permission from the photographer to publish the photo(s) under our CC BY 4.0 license.

The images shown in 1A are taken by Soomin Cho.

6) We note that your Data Availability Statement is currently as follows: "All relevant data are within the manuscript and its Supporting Information files.". Please confirm at this time whether or not your submission contains all raw data required to replicate the results of your study. Authors must share the “minimal data set” for their submission. PLOS defines the minimal data set to consist of the data required to replicate all study findings reported in the article, as well as related metadata and methods (https://journals.plos.org/plosone/s/data-availability#loc-minimal-data-set-definition).
For example, authors should submit the following data:
1) The values behind the means, standard deviations and other measures reported;
2) The values used to build graphs;
3) The points extracted from images for analysis..
Authors do not need to submit their entire data set if only a portion of the data was used in the reported study.
If your submission does not contain these data, please either upload them as Supporting Information files or deposit them to a stable, public repository and provide us with the relevant URLs, DOIs, or accession numbers. For a list of recommended repositories, please see https://journals.plos.org/plosone/s/recommended-repositories.
If there are ethical or legal restrictions on sharing a de-identified data set, please explain them in detail (e.g., data contain potentially sensitive information, data are owned by a third-party organization, etc.) and who has imposed them (e.g., an ethics committee). Please also provide contact information for a data access committee, ethics committee, or other institutional body to which data requests may be sent. If data are owned by a third party, please indicate how others may request data access.

An Excel file with the values used to generate all the graphs is uploaded as Supplementary Table S1.

7) Please amend your detailed Financial Disclosure statement. This is published with the article. It must therefore be completed in full sentences and contain the exact wording you wish to be published.
1) State what role the funders took in the study. If the funders had no role in your study, please state: "The funders had no role in study design, data collection and analysis,

decision to publish, or preparation of the manuscript."
2) If any authors received a salary from any of your funders, please state which authors and which funders..
If you did not receive any funding for this study, please simply state: u201cThe authors received no specific funding for this work.u201d

Modified as suggested.

8) Please ensure that the funders and grant numbers match between the Financial Disclosure field and the Funding Information tab in your submission form. Note that the funders must be provided in the same order in both places as well.

Done.

9) Please ensure that the affiliations of the authors listed on the manuscript title page (CITY) do exactly match with the affiliations provided in the online submission form
NOTE: Affiliations should include a department (if applicable), an institution, a CITY, and a country.

Done.

Please indicate by return email the full and correct funding information for your study and confirm the order in which funding contributions should appear.

The order listed in the Funding section of the manuscript and the Funding Information tab of the online submission system is correct.
